# Performance of the plasma Aβ42/Aβ40 ratio, measured with a novel HPLC-MS/MS method, as a biomarker of amyloid PET status in a DPUK-KOREAN cohort

**DOI:** 10.1186/s13195-021-00911-7

**Published:** 2021-10-22

**Authors:** Hyemin Jang, Ji Sun Kim, Hye Joo Lee, Chi-Hun Kim, Duk L. Na, Hee Jin Kim, José Antonio Allué, Leticia Sarasa, Sergio Castillo, Pedro Pesini, John Gallacher, Sang Won Seo, on behalf of DPUK

**Affiliations:** 1 Department of Neurology, Samsung Medical Center, Sungkyunkwan University School of Medicine, 81 Irwon-ro, Gangnam-gu, Seoul, 06351 Republic of Korea; 2 Neuroscience Center, Samsung Medical Center, 81 Irwon-ro, Gangnam-gu, Seoul, 06351 Republic of Korea; 3 Alzheimer’s Disease Convergence Research Center, Samsung Medical Center, 81 Irwon-ro, Gangnam-gu, Seoul, 06351 Republic of Korea; 4 Department of Neurology, School of Medicine, Kyungpook National University, Kyungpook National University Chilgok Hospital, Daegu, South Korea; 5 Department of Psychiatry, University of Oxford, Warneford Hospital, Oxford, UK; 6 Stem Cell & Regenerative Medicine Institute, Samsung Medical Center, 81 Irwon-ro, Gangnam-gu, Seoul, 06351 Republic of Korea; 7 Department of Health Sciences and Technology, Seoul, Republic of Korea; 8 Araclon Biotech-Grifols, Vía Hispanidad, 21, 50009 Zaragoza, Spain; 9 Department of Clinical Research Design & Evaluation, SAIHST, Sungkyunkwan University, 81 Irwon-ro, Gangnam-gu, Seoul, 06351 Republic of Korea; 10 Department of Intelligent Precision Healthcare Convergence, Sungkyunkwan University School of Medicine, Seoul, Republic of Korea

**Keywords:** Biomarker, Alzheimer’s disease, Amyloid, Plasma, Aβ42/Aβ40, Liquid chromatography-mass spectrometry

## Abstract

**Background:**

We assessed the feasibility of plasma Aβ42/Aβ40 determined using a novel liquid chromatography-mass spectrometry method (LC-MS) as a useful biomarker of PET status in a Korean cohort from the DPUK Study.

**Methods:**

A total of 580 participants belonging to six groups, Alzheimer’s disease dementia (ADD, *n* = 134), amnestic mild cognitive impairment (aMCI, *n* = 212), old controls (OC, *n* = 149), young controls (YC, *n* = 15), subcortical vascular cognitive impairment (SVCI, *n* = 58), and cerebral amyloid angiopathy (CAA, *n* = 12), were included in this study. Plasma Aβ40 and Aβ42 were quantitated using a new antibody-free, LC-MS, which drastically reduced the sample preparation time and cost. We performed receiver operating characteristic (ROC) analysis to develop the cutoff of Aβ42/Aβ40 and investigated its performance predicting centiloid-based PET positivity (PET+).

**Results:**

Plasma Aβ42/Aβ40 were lower for PET+ individuals in ADD, aMCI, OC, and SVCI (*p* < 0.001), but not in CAA (*p* = 0.133). In the group of YC, OC, aMCI, and ADD groups, plasma Aβ42/Aβ40 predicted PET+ with an area under the ROC curve (*AUC*) of 0.814 at a cutoff of 0.2576. When adding age, *APOE*4, and diagnosis, the *AUC* significantly improved to 0.912.

**Conclusion:**

Plasma Aβ42/Aβ40, as measured by this novel LC-MS method, showed good discriminating performance based on PET positivity.

**Supplementary Information:**

The online version contains supplementary material available at 10.1186/s13195-021-00911-7.

## Introduction

Alzheimer’s disease (AD) is characterized by amyloid β (Aβ) deposition in the brain. Due to the rapid development of Aβ biomarker testing, earlier diagnosis of AD has become possible. Currently, Aβ positron emission tomography (PET) and cerebrospinal fluid (CSF) are the most validated methods for detection of the fibrillary and soluble forms of Aβ, respectively [1]. However, as these methods have limitations in terms of cost, accessibility, exposure to radiation, and invasiveness, the need for blood biomarkers that are more easily accessible has been increasing. Some studies have attempted to prove the clinical utility of plasma Aβ biomarker by investigating its correspondence with Aβ PET and CSF [2–11]. Particularly, recent studies that used immunoprecipitation-mass spectrometry (IP-MS) to measure plasma Aβ42/Aβ40 showed promising results with high predictive accuracy for PET positivity [5, 8, 12].

Although the exact physiology underlying the pathological production and clearance of plasma Aβ remains unclear, there is increasing evidence that Aβ in the brain is cleared via transport into the plasma through the blood-brain barrier (BBB) or CSF [13]. In terms of the BBB transport of Aβ, there are several factors that have been known to affect it. Increased age is associated with impaired BBB transport of Aβ [14]. Depending on the genotype, *APOE* protein is also known to affect Aβ transport by combining differently with Aβ and lipoprotein receptor-related protein [15, 16]. Changes in Aβ fluid biomarkers may occur earlier than Aβ PET in the preclinical phase of the AD continuum [17, 18], as it is known that the dysregulated kinetics of soluble Aβ precedes Aβ deposition in the brain [19]. In fact, previous studies have shown that most discordant cases in preclinical AD were CSF+/PET− [17]. Although it has not been as thoroughly investigated as CSF, it would be reasonable to expect that most discordant cases in preclinical AD might also be plasma+/PET−. However, considering that Aβ kinetics in the plasma Aβ level changes as the disease progresses [5], the association of plasma Aβ and Aβ deposition in the brain might vary depending on the three cognitive stages: normal control, mild cognitive impairment (MCI), and dementia. Thus, we can assume that age, *APOE* genotype, and the cognitive stage might affect the relationship between the plasma Aβ level and Aβ deposition detected on PET.

Subcortical vascular cognitive impairment (SVCI) is characterized by extensive cerebrovascular diseases (CVD), such as white matter hyperintensities (WMH) and lacunes on MRI. CVD is an important clinical factor that could damage BBB through various mechanisms such as ischemia, oxidative stress, and inflammation. Subsequently, these BBB damages may affect Aβ clearance, which in turn may result in plasma Aβ changes. In fact, previous studies have reported that WMH is associated with plasma Aβ40 or Aβ42 [3, 20]. Thus, patients with SVCI might show a different performance from patients on the AD continuum in their plasma Aβ42/Aβ40, which predicts Aβ PET positivity.

In the present study, we aimed to investigate the predictive accuracy of plasma Aβ42/Aβ40, using a specific liquid chromatography-MS (LC-MS) method in a large-sized cohort which included various cognitive stages and etiologies. First, we performed a 10-fold cross-validation in patients across the AD continuum. We hypothesized that the plasma Aβ42/Aβ40 would successfully predict PET positivity and that the predictive performance would increase if age, *APOE* genotype, and cognitive stage were included in the model. Then, to investigate whether CVD may affect the predictive performance of plasma Aβ42/Aβ40 for PET positivity, we also validated our model in patients with SVCI.

## Materials and methods

### Subjects

This study cohort of 580 participants represents a sample obtained from the memory clinic at Samsung Medical Center between 2017 and 2019. The selection process is shown in Supplementary Figure 1. All participants underwent brain magnetic resonance imaging (MRI) and Aβ PET with either ^18^F-florbetaben (FBB) or ^18^F-flutemetamol (FMM) [21]. The study participants were divided into six diagnostic groups: young controls (YC, cognitively unimpaired individuals younger than 45 years), old controls (OC, cognitively unimpaired individuals older than 45 years), amnestic mild cognitive impairment (aMCI), Alzheimer’s disease dementia (ADD), SVCI, and CAA. All participants, except the YCs, underwent detailed neuropsychological tests called the Seoul Neuropsychological Screening Battery (SNSB) [21]. YCs (*n* = 15) were healthy volunteers without a history of neurological or psychiatric disorders. OCs (*n* = 149) were defined to have normal cognition on neuropsychological tests (above the −1.0 standard deviation (SD) of age- and education-matched norms in memory and −.1.5 SD in other cognitive domains [22]) without a history of neurological or psychiatric disorders. aMCI (*n* = 212) was defined using Petersen’s criteria for MCI [23]. Probable ADD (*n* = 134) [24] was defined using the National Institute of Neurological and Communicative Disorders and Stroke and the Alzheimer’s disease and Related Disorders Association criteria [25]. Those with aMCI or ADD had WMH that were either minimal (periventricular WMH < 5 mm and deep WMH < 5 mm) or moderate (between minimal and severe WMH classifications) based on the Fazekas ischemia criteria [26]. SVCI (*n* = 58, subcortical vascular MCI and subcortical vascular dementia) was defined when patients met all three of the following criteria: (1) subjective cognitive complaints by the patient or caregiver; (2) objective cognitive impairment below the 16th percentile in any domain including language, visuospatial, memory, or frontal function on neuropsychological tests; and (3) severe ischemia on brain MRI, defined as periventricular WMH ≥ 10 mm and deep WMH ≥ 25 mm, as modified from the Fazekas ischemia criteria [26]. CAA (*n* = 12) was defined when the individuals met the modified Boston criteria for probable CAA [27–29] regardless of cognitive status.

All participants were assessed through clinical interviews and neurologic examinations, and clinical diagnoses were established by consensus among a multidisciplinary team. Blood tests included complete blood count, blood chemistry tests, vitamin B12/folate measurement, syphilis serology, thyroid function test, and *APOE* genotyping. Patients were excluded if they had territorial infarctions, cortical stroke, brain tumor, or vascular malformation on MRI. Patients with WMH due to radiation injury, multiple sclerosis, vasculitis, or leukodystrophy were also excluded.

This study was approved by the institutional review board of Samsung Medical Center.

### Plasma collection and processing

We obtained 8 mL of blood from each participant and placed into a 0.5 M EDTA-containing tube and mixed it for 5 min. The Green Cross lab picked up the samples that were stocked in the cooler after mixing. Plasma was extracted from the blood sample after a 10-min centrifuge (1300 g) and dispensed into 5 or 10 vials at a volume of 0.3 mL each. All plasma samples were kept frozen at −75 °C until LC-MS analysis. The process complied with the manual for human resource collection and registration of the National Biobank of the Republic of Korea [30]. The median interval between plasma collection and Aβ PET scans was 0.5 days (interquartile range, 0–37.5 days).

### Liquid chromatography-mass spectrometry (LC-MS)

The prepared plasma samples were sent to Araclon Biotech (Zaragoza, Spain) and analyzed using LC-MS. Plasma samples were analyzed using a novel antibody-free liquid chromatography-differential mobility spectrometry-triple quadrupole mass spectrometry (HPLC-DMS-MS/MS) method. The analytical platform was composed of a QTRAP 6500+ hybrid linear ion trap-triple quadrupole mass spectrometer fitted with a differential mobility spectrometry interface (SelexION) and coupled to an M3 Micro LC system (Sciex, Framingham, MA, USA). Samples (200 μL each) were analyzed singles. Analytes were extracted directly from plasma, and no immunoprecipitation procedure was performed. Intact Aβ40 and Aβ42 species were analyzed as no enzymatic digestion was performed. The specifics of the method are the subject matter of patent application (EP2020382352) that will be publicly available for inspection within 18 months of its filing date.

### Analysis of mass spectrometry data

Calibration curves were prepared in human plasma after spiking ^15^N-Aβ40 and ^15^N-Aβ42 at seven concentration levels. Quality control samples were also prepared in human plasma at three concentration levels (low: 3 × LLOQ, mid, and high). The calibration ranges were 50–1000 pg/mL for ^15^N-Aβ40 and 10–200 pg/mL for ^15^N-Aβ42. The LLOQ for ^15^N-Aβ40 was 50 pg/mL (% relative error RE = 0.3% and coefficient of variation CV = 7%). The LLOQ for ^15^N-Aβ42 was 10 pg/mL (RE = −1.5% and CV = 11%).

Two calibration curves were used in each analytical run, one at the beginning and one at the end of the sequence. Additionally, six quality control samples, uniformly distributed along the sequence, were analyzed in each run.

Deuterated internal standards (^2^H-Aβ40 and ^2^H-Aβ42) were spiked in all samples (calibration curves, quality control, and study samples). Response ratios corresponding to endogenous species in the study samples (^14^N-Aβ40/^2^H-Aβ40 and ^14^N-Aβ42/^2^H-Aβ42) were interpolated in the calibration curves made with ^15^N analogues. Suitability test samples were analyzed every day at the beginning of the analytical run to evaluate system performance and equal transmission for light (^14^N) and heavy (^15^N) species.

Analyst 1.6.3. Software (Sciex) was used for data acquisition, and the MultiQuant 3.0.3. software (Sciex) was used for data processing.

Eight participants (one OC, two with aMCI, four with ADD, and one with SVCI) with Aβ40 values below the lower limit of quantification (<LLQ) were excluded from the analysis.

### Brain MRI

All participants underwent standardized T2, 3-dimensional T1 turbo field echo images, 3-dimensional (3D) fluid-attenuated inversion-recovery (FLAIR), and T2×-weighted gradient echo (GRE)-MRIs at Samsung Medical Center using a 3.0T MRI scanner (Philips 3.0T Achieva; Philips Healthcare, Andover, MA, USA) [31]. The following parameters were used for the T2* GRE images: axial slice thickness 5.0 mm, inter-slice thickness 2 mm, repetition time (TR) 669 ms, echo time (TE) 16 ms, flip angle 18°, and matrix size 560 × 560 pixels. We acquired 3D T1 images with the following parameters: sagittal slice thickness 1.0 mm, over contiguous slices with 50% overlap, TR 9.9 ms, TE 4.6 ms, flip angle 8°, and matrix size 240 × 240 pixels, reconstructed to 480 × 480 over a field of view of 240 mm. 3D FLAIR images were obtained with the following parameters: axial slice thickness 2 mm, no gap, TR 11,000 ms, TE 125 ms, flip angle 90°, and matrix size 512 × 512 pixels.

### Aβ PET imaging acquisition, analysis, centiloid, and definition of Aβ positivity

All participants underwent either FBB or FMM PET at Samsung Medical Center using a Discovery STe PET/CT scanner (GE Medical Systems, Milwaukee, WI, USA) in 3D scanning mode that examined 47 slices of 3.3-mm thickness spanning the entire brain [32, 33]. CT images were acquired using a 16-slice helical CT (140 KeV, 80 mA; 3.75-mm section width) for attenuation correction. According to the protocols proposed by the ligands’ manufacturers, a 20-min emission PET scan with dynamic mode (consisting of 4 × 5 min frames) was performed 90 min after injection of a mean dose of 311.5 MBq of FBB or 185 MBq of FMM. 3D PET images were reconstructed in a 128 × 128 × 48 matrix with a voxel size of 2 mm × 2 mm × 3.27 mm using the ordered-subsets expectation maximization algorithm (FBB iterations = 4 and subset = 20; FMM iterations = 4 and subset = 20).

PET images were co-registered to individual MRIs normalized to a T1-weighted MNI-152 template using SPM8 through MATLAB2014b (Mathworks, Natick, MA, USA). After standard space registration, the brain was divided into 116 gray matter regions using the Automated Anatomical Labeling atlas [34]. The global standard uptake value ratio (SUVR) was obtained with reference to the whole cerebellum to quantify FBB and FMM retention, from the volume-weighted average SUVR of 28 bilateral cerebral cortical volumes-of-interest (VOIs) [32, 35]. The SUVR cutoff value for Aβ positivity was calculated using the iterative outlier approach [36, 37], which removes cases from cognitively normal participants over 55 years of age until all outliers are excluded. Once all outliers are removed from the dataset, 2.5% is added to the SUVR of the highest remaining case, which results in a cutoff value [36]. As a result, the cortical SUVR cutoff values were 1.1 for FBB and 1.03 for FMM, respectively, when the whole cerebellum was used as a reference region [37, 38].

In our previous study, we directly converted the SUVR values of the FBB or FMM CTX VOI into direct comparison centiloid units (dcCL) using the dcCL conversion equation [39, 40]. One AD patient (with Aβ40 < LLQ) and one CAA patient were excluded due to imaging analysis errors. To obtain the dcCL cutoff value for Aβ positivity, we performed receiver operating characteristic (ROC) analysis using Aβ positivity based on the SUVR cutoff for each PET as the standard of truth. Therefore, the dcCL cutoff value was 25.11 with an area under the curve (*AUC*) of 0.994, and we defined Aβ positivity using this dcCL cutoff in this study (Supplementary Figure 2).

### Statistical analyses

We compared plasma Aβ40, Aβ42, and Aβ42/Aβ40 between Aβ PET-positive and PET-negative participants in each diagnostic group using the Mann-Whitney *U* test. We investigated the correlation between plasma Aβ42/Aβ40 and dcCL units using Spearman’s correlation analysis. We then performed logistic regression followed by ROC analyses to establish the performance of Aβ42/Aβ40 predicting dcCL-based Aβ PET positivity (unadjusted model) in the AD continuum. The Aβ42/Aβ40 cutoff was defined as the value that gives the maximum Youden index (sensitivity + specificity − 1) from this ROC analysis. We then calculated the positive percent agreement (PPA), which was defined as the proportion of dcCL-based PET-positive participants that are plasma positive, and negative percent agreement (NPA), which was defined as the proportion of dcCL-based PET-negative participants that were plasma negative. We defined plasma Aβ42/Aβ40 as abnormal (plasma+) when it was lower than the cutoff value. Therefore, the concordance rate of Aβ PET and plasma biomarker results was calculated as the number of plasma+/PET+ plus plasma−/PET− cases over the total number of participants in the analysis.

To evaluate whether concordance rate differs according to different Aβ PET positivity cutoffs, we performed a sensitivity analysis using two Aβ dcCL cutoffs: (1) the first cutoff of 24.6 was obtained from the ROC analysis using amyloid positivity based on visual reading (instead of SUVR cutoff) as the standard of truth, and (2) the second cutoff of 20 was arbitrarily selected, given that this is a cutoff for the presence of at least moderate plaque density based on a previous pathology study [41].

We performed ROC analyses using a 10-fold cross-validation in AD continuum including YC, OC, aMCI, and ADD to increase the reliability of the model for predicting dcCL-based Aβ PET positivity with different combinations of variables: age, presence of *APOE*4 (either heterozygotes or homozygotes), and cognitive stage (diagnosis). We included diagnosis variables in the adjusted model, as we considered that plasma Aβ42/Aβ40 might be differentially affected by disease status, and the diagnosis group was included in the model as a categorical variable (dummy variables: YC, OC, aMCI, and ADD). The *AUC* of multiple models was compared using the Delong method. Finally, we applied the prediction model to the SVCI cohort for the validation process. All analyses were conducted using the STATA version 15. Statistical significance was set at *p* < 0.05.

### Data availability

Anonymized data for our analyses presented in this report are available upon request from the corresponding authors.

## Results

### Characteristics of the participants

The detailed demographics and clinical characteristics of the 580 participants are presented in Table 1. The mean age of participants (*n* = 580) was 69.8 ± 11.0 years (range, 26–97 years). Females accounted for 62.4%, and the frequency of *APOE*4 carriers was 36.9%. All patients underwent Aβ PET, and Aβ positivity by the dcCL cutoff was 48.4%. Aβ positivity was 85.0% for ADD, 56.1% for aMCI, 11.4% for OC, 37.9% for SVCI, and 81.8% for CAA. No YC scans showed PET+ scans. The demographics of the study participants according to the diagnosis and PET positivity are shown in Supplementary Table 1.

**Table 1:** Demographics and clinical characteristics of the study participants

	Total	OC	aMCI	ADD	SVCI	CAA	YC
***N***	580	149	212	134	58	12	15
**Age (years)**	69.8 ± 11.0	69.6 ± 7.7	70.0 ± 8.3	70.2 ± 11.0	77.4 ± 8.5^*,†,$^	76.8 ± 6.9	32.5 ± 3.9^*,†,$^
**Female sex**	362 (62.4)	98 (65.8)	119 (56.1)	90 (67.7)^†^	44 (74.6)^†^	6 (50.0)	6 (40.0)^*,$,^^
***APOE*4 carrier**	214 (36.9)	38 (25.5)	85 (40.1)^*^	75 (56.4)^*,†^	12 (20.3)^†,$^	1 (8.3)^†,$^	3 (20.0)^$^
**PET positivity**^**a**^	280/578^b^ (48.4)	17 (11.4)	119 (56.1)^*^	113/133 (85.0)^*,†^	22 (37.9)^*,†,$^	9/11 (81.8)^*,^^	0 (0.0)^†,$,^,#^
**MMSE**	24 ± 5.4	27.8 ± 2.5	22.5 ± 3.3^*^	18.5 ± 5.3^*,†^	22 ± 5.4^*,†,$^	21 ± 7.3^*,$^	N/A
**Plasma Aβ42**	56.9 ± 16.2 (*n* = 577)	60 ± 14.4	56.1 ± 16.3	51.7 ± 15.5^*^	64.8 ± 18.2^†,$^	55.7 ± 20.3	52.2±5^^^
**Plasma Aβ40**	217 ± 58.2 (*n* = 572)	215.8 ± 52.5	216 ± 55	210.8 ± 60.3	241.7 ± 68.1^*,†,$^	237.1 ± 86.4	184.4 ± 15.6^^^
**Plasma Aβ42/Aβ40 **	0.27 ± 0.06 (*n* = 572)	0.28 ± 0.05	0.27 ± 0.05^*^	0.26 ± 0.08^*^	0.28 ± 0.07	0.24 ± 0.03	0.28 ± 0.03

Values are presented as mean ± standard deviation or number (%) as appropriately

*ADD* Alzheimer’s disease dementia; *aMCI* amnestic mild cognitive impairment; *OC* old controls; *SVCI* subcortical vascular cognitive impairment; *CAA* cerebral amyloid angiopathy; *YC* young controls

^a^PET positivity is based on the centiloid unit

^b^Two patients were excluded due to image processing errors

^*^p < 0.05 compared to OC

^†^*p* < 0.05 compared to aMCI

^$^*p* < 0.05 compared to ADD

^^^*p* < 0.05 compared to SVCI

^#^*p* < 0.05 compared to CAA

### Comparison between plasma Aβ biomarkers and Aβ uptakes on PET

**Fig. 1:**
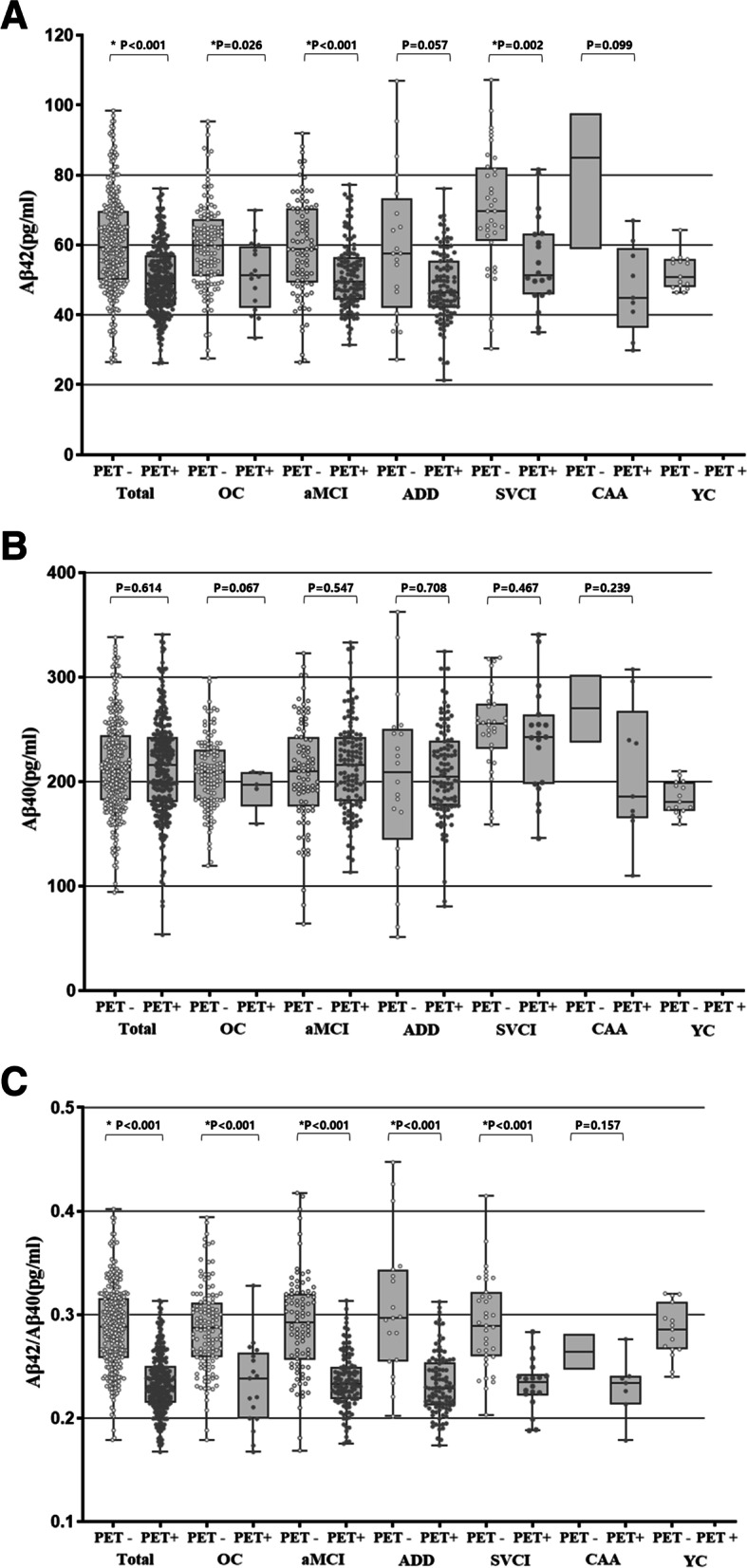
**A** Plasma Aβ42, **B** Aβ40, and **C** Aβ42/Aβ40 according to Aβ positivity on PET in each diagnostic group. This box-and-whisker plot shows a box with a lower edge at the lower quartile (25%), upper edge at the upper quartile (75%), the middle of the box at the median, and the maximum and minimum as whiskers. Abbreviations: OC, old controls; aMCI, amnestic mild cognitive impairment; ADD, Alzheimer’s disease dementia; SVCI, subcortical vascular cognitive impairment; CAA, cerebral amyloid angiopathy; YC, young controls; PET, positron emission tomography

Figure 1 shows plasma Aβ42 (A), Aβ40 (B), and Aβ42/Aβ40 (C) according to Aβ PET positivity in the total group and in each diagnostic group. Plasma Aβ42 levels were significantly lower in the PET-positive groups than in the PET-negative groups, except for the ADD and CAA groups (total, aMCI *p* < 0.001; OC, *p* = 0.026; ADD, *p* = 0.057; SVCI, *p* = 0.002; CAA, *p* = 0.099) (Fig. 1A). Aβ40 was not significantly different between PET-positive and PET-negative groups (Fig. 1B). In all diagnostic groups except for CAA, Aβ PET-positive groups had significantly lower plasma Aβ42/Aβ40 levels than Aβ PET-negative groups (total, OC, aMCI, ADD, and SVCI, *p* < 0.001; CAA, *p* = 0.157) (Fig. 1C).

**Fig. 2:**
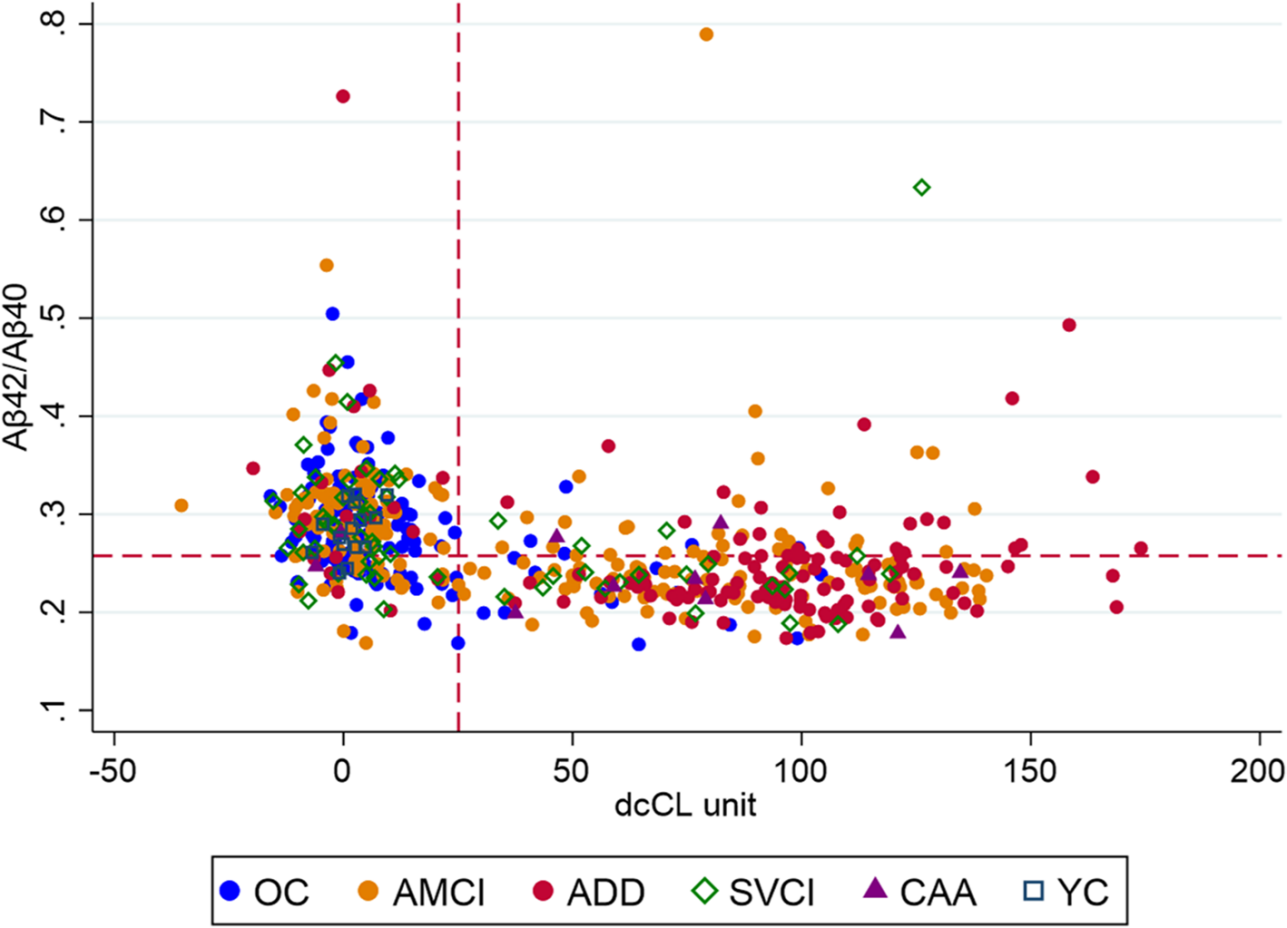
Scatterplot of plasma Aβ42/Aβ40 and amyloid PET centiloid and their correlation. The cutoff value of PET centiloid and Aβ42/Aβ40 were 25.11 and 0.2576, respectively. Abbreviations: OC, old controls; aMCI, amnestic mild cognitive impairment; ADD, Alzheimer’s disease dementia; SVCI, subcortical vascular cognitive impairment; CAA, cerebral amyloid angiopathy; YC, young controls; PET, positron emission tomography

In the total group, baseline plasma Aβ42/Aβ40 was inversely correlated with continuous dcCL units with a Spearman’s rho of −0.508 (*p* < 0.001) (Fig. 2).

### Concordance of plasma Aβ42/Aβ40 and Aβ positivity on PET

**Fig. 3:**
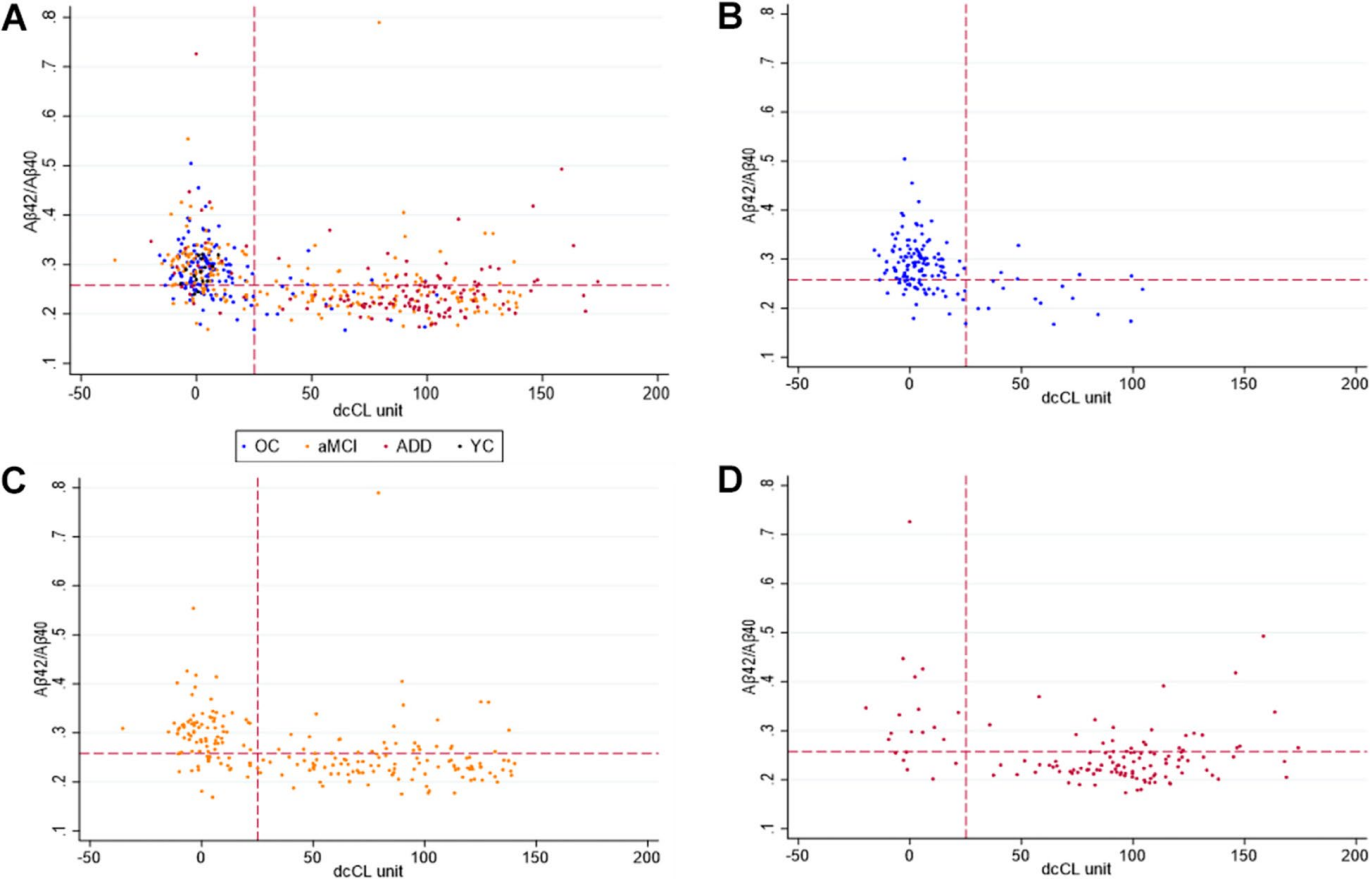
Scatterplot of plasma Aβ42/Aβ40 and Aβ PET centiloid in the **A** total, **B** OC, **C** aMCI, and **D** ADD groups. The dashed red lines indicate cutoffs for plasma Aβ42/Aβ40 (0.2576) based on the maximum Youden index and for Aβ PET centiloids (25.11) for amyloid PET positivity. The cutoff value of PET centiloid and Aβ42/Aβ40 were 25.11 and 0.2576, respectively. Abbreviations: total, total participants excluding YCs; OC, old controls; aMCI, amnestic mild cognitive impairment; ADD, Alzheimer’s disease dementia; SVCI, subcortical vascular cognitive impairment; CAA, cerebral amyloid angiopathy; YC, young controls; PET, positron emission tomography

We performed ROC analysis in the YC, OC, aMCI, and ADD groups. The ROC analysis demonstrated that plasma Aβ42/Aβ40 alone was a good predictor of Aβ PET dcCL-based positivity, with an *AUC* of 0.814 (unadjusted model). The plasma Aβ42/Aβ40 cutoff with the maximum Youden index was 0.2576 and yielded a PPA of 75.8% and NPA of 76.7%. Using this cutoff, a good concordance rate between plasma Aβ42/Aβ40 and PET positivity was achieved (384/503 = 76.3%). The remaining 119 patients with discordant positivity included 60 plasma+/PET− and 59 plasma−/PET+ patients. When the cutoff was applied to all diagnostic groups, each group showed a high concordance rate (72.7 to 86.7%). In terms of discordant cases, OC showed more plasma+/PET− (*n* = 29) than plasma−/PET+ (*n* = 5) (*p* < 0.001), while ADD showed more plasma−/PET+ (*n* = 25) than plasma+/PET− (*n* = 6) (*p* < 0.001). However, the percentage of plasma+/PET− (*n* = 23) and plasma−/PET+ (*n* = 29) participants were not different in the aMCI group (*p* = 0.129) (Table 2, Fig. 3). When we investigated this discordance pattern with different PET positivity definitions using different CL cutoff values (24.6 and 20), the results remained similar as OC showed more plasma+/PET− and ADD showed more plasma−/PET+ cases (Supplementary Table 2).

**Table 2:** Performance of plasma Aβ42/Aβ40 to predict PET positivity and concordance with PET

	***AUC*** (unadjusted)	***AUC*** (adjusted)	Concordant/total cases^**a**^ (concordance rate)	Discordant cases^**a**^	
				Plasma+/PET−	Plasma−/PET+
Total	0.814	0.920^b^	384/503 (76.3%)	60 (11.9%)	59 (11.7%)
OC	0.826	0.890^c^	114/148 (77.0%)	29 (19.6%)	5 (3.4%)
aMCI	0.815	0.872^c^	158/210 (75.2%)	23 (11.0%)	29 (13.8%)
ADD	0.812	0.877^c^	99/130 (72.7%)	6 (4.6%)	25 (19.2%)
YC	NA	NA	13/15 (86.7%)	2 (13.3%)	NA

*PET* positron emission tomography; *ADD* Alzheimer’s disease dementia; *aMCI* amnestic mild cognitive impairment; *OC* old controls; *YC* young controls; *AUC* area under the receiver operating characteristic curve

^a^This concordance classification was based on the plasma Aβ42/Aβ40 ratio of 0.2576 obtained from the total group

^b^Logistic analysis after adjusting for age, *APOE*4 status, and diagnosis

^c^Logistic analysis after adjusting for age and *APOE*4 status

We also performed ROC analyses for each diagnostic group. Particularly in the OC group, plasma Aβ42/Aβ40 alone predicted PET positivity. The *AUC* of 0.826 (PPA 94.1, NPA 62.6) plasma Aβ42/Aβ40 alone predicted PET positivity with good performance in all groups (*AUC*s, 0.812–0.826), which significantly increased in the age- and *APOE*4-adjusted models (*AUC*s 0.872 to 0.890) (Table 2).

### Comparison of prediction models including different combinations of variables

**Fig. 4:**
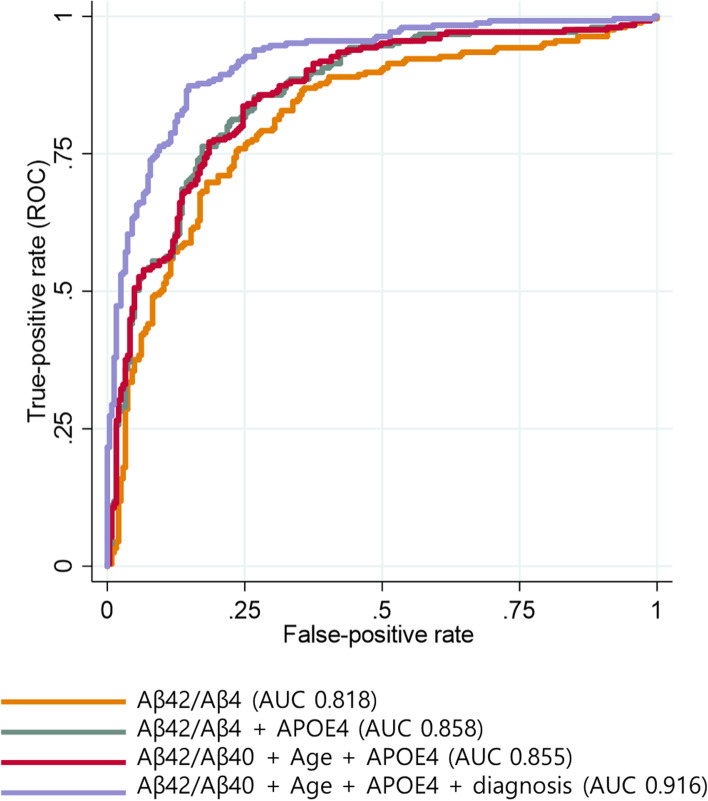
Receiver operating characteristic curves for Aβ42/Aβ40 and Aβ42/Aβ40 plus covariates for predicting amyloid positivity. Abbreviations: *AUC*, area under the receiver operating characteristic curve; Dx, diagnosis

We compared multiple models with different combinations of variables to evaluate whether adding plasma Aβ42/Aβ40 to the clinical variables increased predictive performance for Aβ positivity. We performed a 10-fold cross-validation in AD continuum to obtain the *AUC*s. As shown in Table 3, the *AUC* of the age and *APOE*4 model (model 3, Fig. 4) was 0.727, and it improved to 0.855 when Aβ42/Aβ40 was added (model 6). When we added the diagnosis group (cognitive stage) in addition to age, *APOE*4 status, and plasma Aβ42/Aβ40 in the model (model 7) to predict Aβ PET positivity, the *AUC* improved to 0.916. Except for model 2 vs. model 3 (*p* = 0.661) and model 5 vs. model 6 (*p* = 0.759), both individual models in Table 3 show statistically different *AUC* values (all *p* < 0.05) (Fig. 3).

**Table 3:** Models with different combinations of variables

	Model	10-fold cross-validation in AD continuum	Validation in the SVCI group
Model 1	Age	0.506 (0.407, 0.512)	0.643 (0.485, 0.8)
Model 2	*APOE*4	0.723 (0.614, 0.717)	0.59 (0.475, 0.705)
Model 3	Age + *APOE*4	0.727 (0.652, 0.751)	0.699 (0.558, 0.839)
Model 4	Aβ42/Aβ40	0.818 (0.76, 0.847)	0.823 (0.702, 0.944)
Model 5	*APOE*4 + Aβ42/Aβ40	0.858 (0.799, 0.876)	0.775 (0.646, 0.905)
Model 6	Age + *APOE*4 + Aβ42/Aβ40	0.855 (0.797, 0.875)	0.777 (0.648, 0.905)
Model 7	Age + *APOE*4 + Aβ42/Aβ40 + diagnosis	0.916 (0.877, 0.934)	NA

Values are presented as *AUC* (95% confidence interval for *AUC*). Except for model 2 vs. model 3 (*p* = 0.661) and model 5 vs. model 6 (*p* = 0.791), all two individual models showed statistically different *AUC* values (all *p* < 0.05)

*AUC* area under the receiver operating characteristic curve

When we applied these models to the SVCI cohort as a validation process, the *AUC* was shown to be as high as 0.823 in model 4 where Aβ42/Aβ40 was the only predictor (Table 3).

## Discussion

In the present study, we investigated the performance of plasma Aβ42/Aβ40 to predict PET positivity using HPLC-MS/MS in a large cohort of carefully phenotyped patients who were on the AD continuum or had SVCI. Our major findings were as follows. First, plasma Aβ42/Aβ40 corresponded well with Aβ PET results of patients on the AD continuum, such as those in the YC, OC, aMCI, and ADD groups. Second, the distribution of plasma+/PET− or plasma−/PET+ among discordant cases differed depending on the cognitive stage of the patients. Third, a 10-fold cross-validation in patients on the AD continuum showed that the predictive performance of plasma Aβ42/Aβ40, when combined with age, *APOE* genotype, and cognitive stage, increased to as much as 0.916. Finally, plasma Aβ42/Aβ40 had a good predictive performance even in the SVCI cohort. Taken together, our findings suggest that Aβ42/Aβ40 in human plasma, as measured by this novel HPLC-MS/MS method, may be useful to screen for Aβ PET positivity across diverse patients and reduce the expense for future clinical trials.

While earlier studies adopting ELISA methods have failed to prove the clinical utility of plasma biomarkers, the most recent study of fully automated plasma immunoassays showed a good performance of plasma Aβ42/Aβ40 with an *AUC* of 0.77 [10]. Especially, a few recent studies using IP-MS methods consistently showed good accuracy in predicting amyloid in the brain across heterogeneous cohorts [5, 8]. However, in our opinion, the HPLC-MS/MS method may provide more advantages compared to IP-MS. The outstanding feature of this method is that it does not involve any immunoprecipitation steps; thus, it is not affected by undesirable effects associated with antibodies such as cross-reactivity and batch-to-batch reproducibility. As it does not involve any additional enzymatic digestion step, intact Aβ40 and Aβ42 levels are quantifiable. Thus, in the present study, we chose to use this novel method to detect plasma Aβ. Our first major finding that plasma Aβ42/Aβ40 corresponded well with Aβ PET results of patients on the AD continuum is supported by the following observations: (1) the plasma Aβ42/Aβ40 levels were significantly different between the PET-positive and PET-negative groups; (2) the plasma Aβ42/Aβ40 levels were well-correlated with quantitative PET uptake measured by dcCL units; and (3) the plasma Aβ42/Aβ40 showed a good *AUC* in predicting Aβ PET results and a high concordance rate. This finding is consistent with previous studies that demonstrated a good performance of plasma biomarkers to predict amyloid PET status, with an *AUC* of 0.75 to 0.9 [5, 8, 10, 12].

Our second major finding was that among discordant cases, there were different distributions of plasma+/PET− or plasma−/PET+ depending on the cognitive stage. Specifically, most of the discordant cases in the OC group were plasma+/PET−, whereas those in the ADD group were plasma−/PET+. The ratio of plasma+/PET− and plasma−/PET+ was similar in the aMCI group. Previously, in terms of plasma, the distribution of discordant cases by cognitive stage had not been investigated extensively. However, previous CSF biomarker studies have shown that most of their discordant cases, especially those with normal cognition, were CSF+/PET−, supporting CSF Aβ as a more sensitive marker of early disease [42–44]. Interestingly, they showed that the number of discordant cases (CSF+/PET−) decreased along the dementia continuum [42, 45], which was congruent with our findings. Similar to CSF Aβ biomarkers, our results suggest that by reflecting soluble Aβ, plasma Aβ42/Aβ40 may be more sensitive in capturing earlier changes in brain β-amyloidosis in the OC group. In contrast, plasma−/PET+ cases were more commonly found in the ADD group, where Aβ plaques were already formed as a major pathology. Considering that Aβ PET changes are more likely to detect the fibrillary form of Aβ [46], it could be suggested that PET and plasma biomarker measures may not be directly interchangeable and instead reflect partially independent processes. However, as the unique discordance profile in ADD has not been observed in previous studies, to replicate our findings, further studies will be needed. In addition, 2 out of the 15 Aβ PET− YCs included in the present study as an absolute control group had a lower Aβ42/Aβ40 (0.240 and 0.244) value compared to the obtained cutoff value of 0.2576. Therefore, such discordant cases should also be followed up for further validation of plasma biomarker testing by HPLC-MS/MS.

Our third major finding was that adding plasma biomarkers to the clinical information had an incremental benefit in terms of predicting PET positivity. As we expected, in a 10-fold cross-validation study based on AD continuum, the predictive performance was improved to 0.916 when age, *APOE*4, and cognitive stage were combined with plasma biomarkers as predictors. Our finding has some clinical implications. Currently, more clinical trials tried to target high-risk but normal cognitive participants, who have Aβ deposition but do not have pronounced neurodegeneration. Under this circumstance, plasma Aβ42/Aβ40, particularly when combined with *APOE* status, could be utilized as the best screening tool because it is inexpensive and easily accessible, and this practical screening test for brain amyloidosis can subsequently reduce the number of amyloid PET scans required for diagnostic confirmation. Specifically, if we assume that the amyloid PET-positive rate is 20% in the elderly over 60 years, and the PPA (sensitivity) of plasma Aβ42/Aβ40 in the OC group is 94.1%, the number needed to screen would be 531, and the number proceeding to scan (plasma+) would be 259 [17]. Therefore, screening participants with plasma Aβ42/Aβ40 could reduce the number of confirmatory amyloid PET scans by 52%.

Our final major finding was that plasma Aβ42/Aβ40 had a good predictive performance even in the SVCI cohort. Previous studies have reported controversial findings between WMH, MRI marker of SVCI, and Aβ fluid biomarkers. While WMH was inversely associated with CSF Aβ isoforms (both Aβ42 and Aβ40) [47, 48], it was positively associated with plasma Aβ40 [3, 49]. Nevertheless, good predictive performance of plasma Aβ42/Aβ40 in the SVCI cohort suggests that plasma Aβ42/Aβ40 could be utilized as a screening tool even in the presence of extensive WMH. Previous studies have reported that CAA has decreased levels of fluid Aβ40 and/or Aβ42 because of vascular deposition of Aβ40 (predominantly), and to a lesser extent Aβ42 [50–55]. We expected that plasma Aβ40 and Aβ42 might also differentially decrease in CAA according to PET positivity; however, we could not find any difference in all three biomarkers between PET+ and PET− patients in the CAA group, although our results might have been underpowered because of the small number of participants. Therefore, a future study with a larger number of CAA participants is needed to investigate the unique changing direction of plasma Aβ40 and the predictive value of Aβ42/Aβ40 for Aβ PET positivity.

### Limitation

Although we included a large cohort of carefully phenotyped participants with a wide array of cognition and etiologies such as CAA or vascular dementia, our study also has several limitations. First, there was no pathologic confirmation to explain the discordant cases of plasma and PET results. In addition, all disease groups were clinically diagnosed; therefore, the presence of other pathologies such as argyrophilic grain disease or hippocampal sclerosis could not be excluded. Second, the number of SVCI and CAA participants was relatively small.

## Conclusion

Nevertheless, we successfully demonstrated the potential clinical utility of plasma biomarkers in memory clinics, using a large number of memory clinic participants in Korea. Our results warrant further validation and development of this plasma Aβ assay, which together with other blood-based biomarkers, could speed up recruitment and reduce screening failure rate and associated costs, opening a new era of clinical trials. Our study also demonstrates the usefulness of a well-developed clinical cohort with research-ready biosamples for advancing AD biomarker studies.

## Supplementary Information

**Additional file 1: Supplementary Figure 1.** Flowchart of sample selection. **Supplementary Figure 2.** Detailed method for amyloid PET imaging analysis and centiloid. **Supplementary Table 1**. Demographics of study participants according to the diagnosis and PET positivity. **Supplementary Table 2.** Demographics of study participants according to the diagnosis and PET positivity

## Data Availability

The data are publicly available and provided upon request.

## Abbreviations

AD: Alzheimer’s disease
ADD: Alzheimer’s disease dementia
aMCI: Amnestic mild cognitive impairment
ADCI: Alzheimer’s disease-related cognitive impairment
OC: Old controls
SVCI: Subcortical vascular cognitive impairment
CAA: Cerebral amyloid angiopathy
YC: Young controls
AUC: Area under the curve
WMH: White matter hyperintensity
LC-MS: Liquid chromatography-mass spectrometry
NPA: Negative percent agreement
PPA: Positive percent agreement
PET: Positron emission tomography
