# Supplementary material for: Performance of the plasma Aβ42/Aβ40 ratio, measured with a novel HPLC-MS/MS method, as a biomarker of amyloid PET status in a DPUK-KOREAN cohort
Source: Alzheimers Res Ther. 2021 Oct 22;13:179. doi: 10.1186/s13195-021-00911-7 (PMC8540152; doi:10.1186/s13195-021-00911-7)
Supplement: Supplementary file 1 — Additional file 1: Supplementary Figure 1. Flowchart of sample selection. Supplementary Figure 2. Detailed method for amyloid PET imaging analysis and centiloid. Supplementary Table 1. Demographics of study participants according to the diagnosis and PET positivity. Supplementary Table 2. Demographics of study participants according to the diagnosis and PET positivity [file 13195_2021_911_MOESM1_ESM.docx]

**Supplementary Figure 1.** Flowchart of sample selection


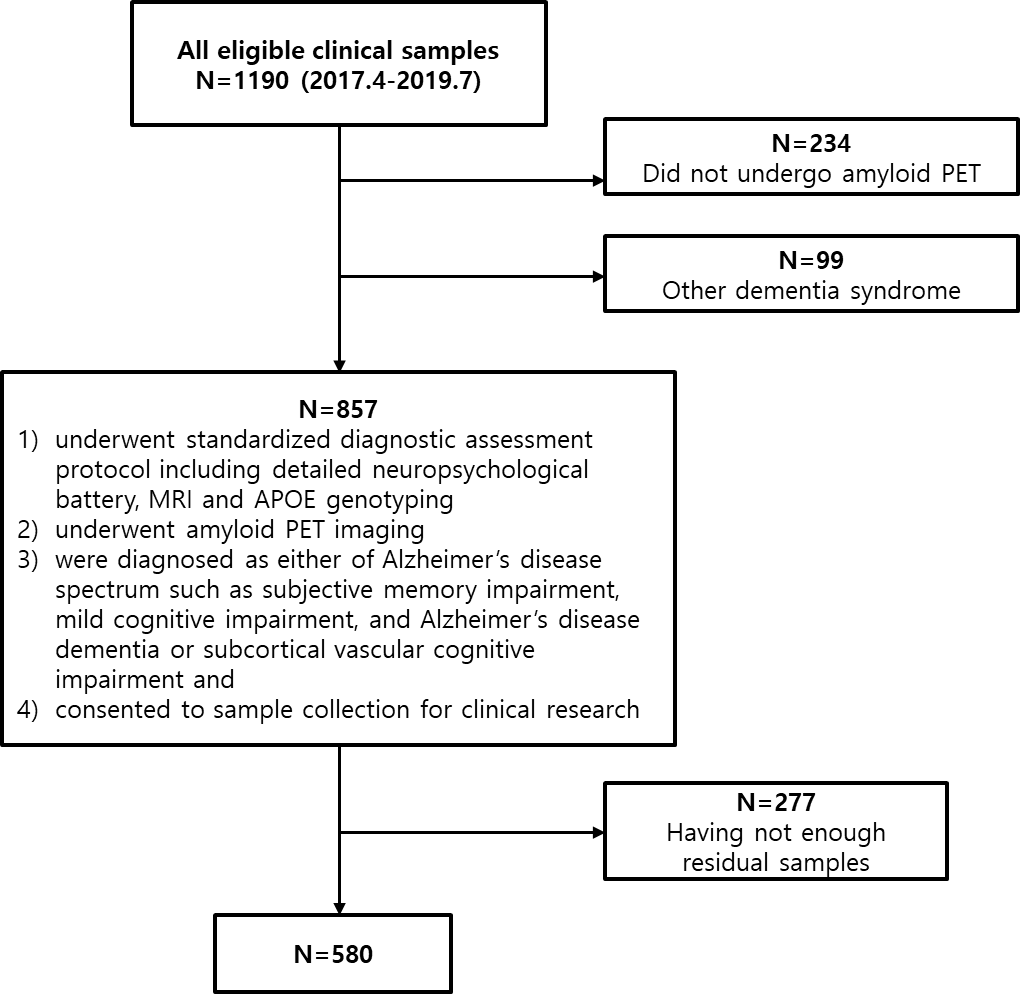


**Supplementary Figure 2.** Detailed method for amyloid PET imaging analysis and centiloid cutoff development


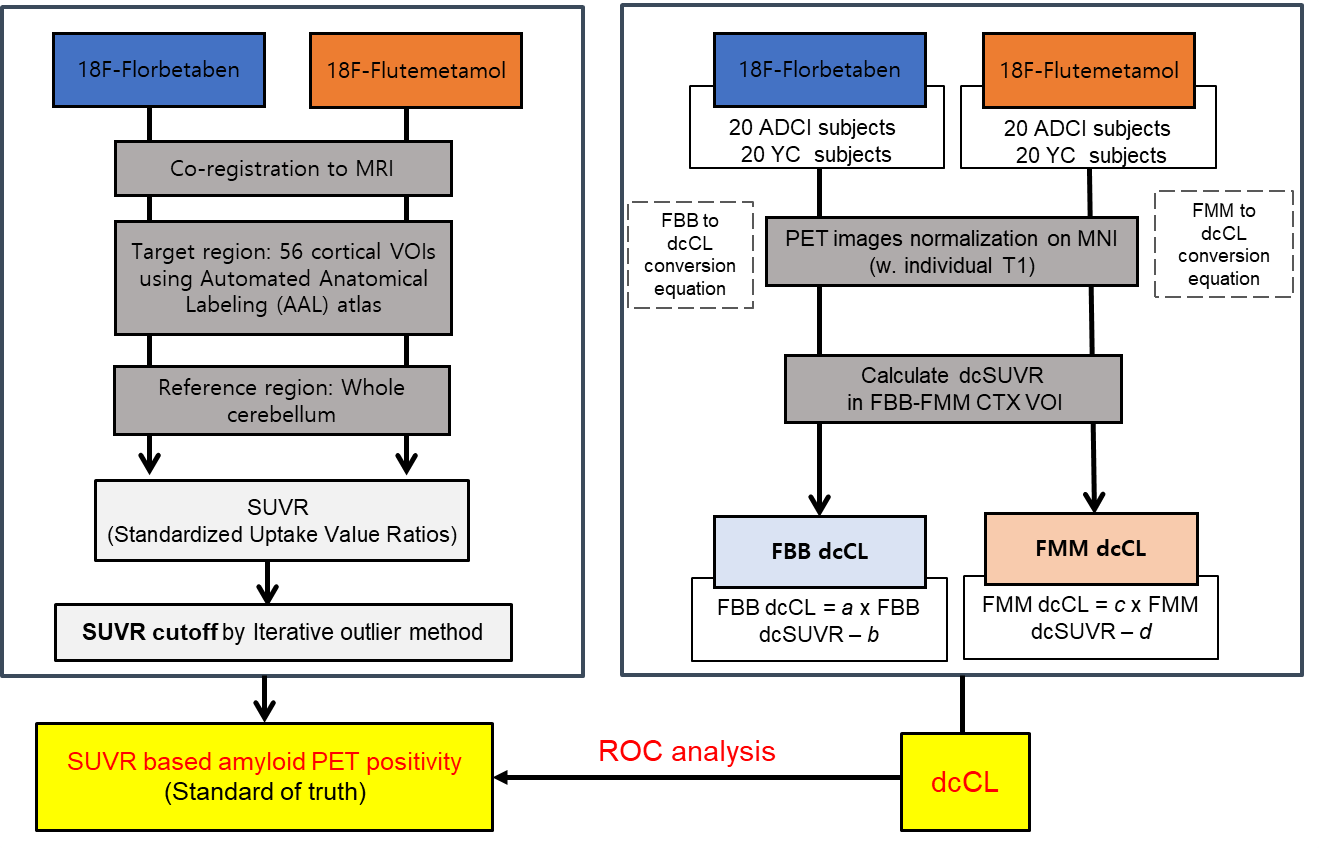


*Abbreviation: ADCI, Alzheimer’s disease cognitive impairment; YC, young controls; dcCL, direct converting centiloid; SUVR, standardized uptake value ratio; PET, positron emission tomography; MRI, magnetic resonance image; ROC, receiver operating characteristic*

**Supplementary Table 1**. Demographics of study participants according to the diagnosis and PET positivity

|  | **Total** |  | **OC** |  | **aMCI** |  | **ADD** |  | **SVCI** |  | **CAA** |  | **YC** |
| --- | --- | --- | --- | --- | --- | --- | --- | --- | --- | --- | --- | --- | --- |
|  | **PET-** | **PET+** | **PET-** | **PET+** | **PET-** | **PET+** | **PET-** | **PET+** | **PET-** | **PET+** | **PET-** | **PET+** |  |
| **N** | 298 | 280 | 132 | 17 | 93 | 119 | 20 | 113 | 36 | 22 | 2 | 9 | 15 |
| **age** | 68.6±12 | 71±9.7* | 68.9±7.7 | 74.5±5.7* | 69.4±8.6 | 70.6±8.2 | 75.6±10.9 | 68.8±11* | 75.7±9 | 79.6±7.3 | 85.5±7.8 | 75±6.3 | 32.5±3.9 |
| **Female** | 183 (61.4) | 177 (63.2) | 88 (66.7) | 10 (58.8) | 47 (50.5) | 72 (60.5) | 13 (65) | 76 (67.3) | 27 (75) | 16 (72.7) | 2 (100) | 3 (33.3) | 6 (40.0) |
| **APOE4 carrier** | 50 (16.8) | 163 (58.2)* | 28 (21.2) | 10 (58.8)* | 11 (11.8) | 73 (61.3)* | 3 (15) | 72 (63.7)* | 5 (13.9) | 7 (31.8) | 0 (0) | 1 (11.1) | 3 (20.0) |
| **MMSE** | 25.9±4.4 | 22±5.6^†^ | 27.8±2.6 | 27.6±1.5 | 26.4±3 | 24.8±3.4^†^ | 18.5±4.8 | 18.4±5.5 | 23.2±4.3 | 20.4±6.7 | 8.5±7.8 | 23.6±5 | N/A |

PET, positron emission tomography; ADD, Alzheimer’s disease dementia; aMCI, amnestic mild cognitive impairment; OC, old normal cognition; SVCI, subcortical vascular cognitive impairment; CAA, cerebral amyloid angiopathy; YC, young normal cognition

T-test or chi-sqaure test were conducted, appropriately

* p<0.05 compared to PET- group

^†^*p* < 0.05 compared PET- group after adjusting for age and education

**Supplementary Table 2.** Demographics of study participants according to the diagnosis and PET positivity

| **Centiloid cutoff** | **Plasma Aβ42/Aβ40 cutoff** | **AUC (unadjusted)** | **Group** | **Concordance rate** | **Discordant cases** | | |
| --- | --- | --- | --- | --- | --- | --- | --- |
|  |  |  |  |  | **PET-/Plasma+** | **PET+/Plasma-** | |
| **25.11** | 0.2576 | 0.920 | Total | 384/503 (76.3%) | 60 (11.9%) | | 59 (11.7%) |
|  |  |  | OC | 114/148 (77.0%) | 29 (19.6%) | | 5 (3.4%) |
|  |  |  | ADD | 99/130 (72.7%) | 6 (4.6%) | | 25 (19.2%) |
| **24.60^a^** | 0.258 | 0.821 | Total | 385/503(76.5%) | 59 (11.7%) | | 59 (11.7%) |
|  |  |  | OC | 115/148 (78%) | 28 (18.9%) | | 5 (3.4%) |
|  |  |  | ADD | 99/130(76.2%) | 6 (4.6%) | | 25 (19.2%) |
| **20^b^** | 0.257 | 0.823 | Total | 384/503(76.3%) | 51 (10.1%) | | 68 (13.5%) |
|  |  |  | OC | 115/148 (78%) | 25 (16.9%) | | 8 (5.4%) |
|  |  |  | ADD | 98/130 (75.4%) | 5 (3.8%) | | 27 (20.8%) |

^a^ Cutoff obtained by ROC analysis visual reading as gold standard

^b^ Cutoff for the presence of at least moderate plaque density based on previous pathology study by Amadoru, S. et al [1]

[1] Amadoru S, Doré V, McLean CA, Hinton F, Shepherd CE, Halliday GM, et al., Comparison of amyloid PET measured in Centiloid units with neuropathological findings in Alzheimer's disease. Alzheimers Res Ther 2020;12:22.
